# Effect of Marination Time on the Antioxidant Properties of Peptides Extracted from Organic Dry-Fermented Beef

**DOI:** 10.3390/biom9100614

**Published:** 2019-10-16

**Authors:** Paulina Kęska, Karolina M. Wójciak, Joanna Stadnik

**Affiliations:** Department of Animal Raw Materials Technology, Faculty of Food Science and Biotechnology, University of Life Sciences in Lublin, Skromna 8, 20-704 Lublin, Poland; paulina.keska@up.lublin.pl (P.K.); joanna.stadnik@up.lublin.pl (J.S.)

**Keywords:** protein, bioactive peptides, acid whey, mass spectrometry

## Abstract

In this study, we evaluated the effect of marination time on changes in the antioxidant properties of peptides extracted from bovine *semimembranosus* muscle. We measured antiradical scavenging capacity and reducing power of the peptides using a spectrophotometric decolorization method; inhibition of lipid oxidation was also assessed by estimating the level of malondialdehyde formed. According to our results, there was no benefit from the doubling of marinating time (from 24 to 48 h) as part of the preprocessing of beef. Samples from S1 batch (24 h marination) showed better antioxidant properties than those from S2 batch. We also tested various color parameters as a reflection of the inhibition of oxidative processes, in which case, the most favorable parameters from the consumer point of view were found to be lightness and redness. The effect of marination time on the degree of proteolytic changes was estimated using peptidomic approach. The degradation of myoglobin, hemoglobin, creatine kinase-type M, and beta-enolase—as the most sensitive proteins to proteolytic degradation—was observed during the 62 days of processing. It seems that the prolongation of marination time as a preprocessing step intensifies the hydrolytic degradation of proteins and peptides during the processing step. This results in the loss (or it has no effect) of antioxidative properties in organic dry-fermented beef.

## 1. Introduction

Marination is a step in the pretreatment of a wide range of food products [1,2]. In the case of meat, it accelerates the process of maturation and it softens and tenderizes the meat in addition to adding a unique flavor to the final product. This is possible by including acidic components in the marinade which loosens the structure of the meat. Marination causes intensive degradation of structural proteins of the meat tissue, e.g., heavy chains of myosin, accelerates therelease of lysosomal enzymes into the cytosol, increases the proteolysis of cathepsin, and decreases the heat stability of perimysial collagen (increased amount of collagen to gelatin conversion at low pH during cooking) [2,3]. According to Berge et al. [3], an injection of 0.5 M lactic acid into the collagen-rich beef muscle (*m. pectoralis profundus*) markedly reduces the toughness and increases the tenderness score of the meat as early as 2 days *postmortem*. Consumers usually introduce marinades into the meat by the method of immersion. In the case of large-scale production, it is necessary to look for a specific marinating agent which is rich in lactic acid bacteria (LAB) and which produces large amounts of lactic acid, which will acidify the environment. These features are fulfilled by the inclusion of acid whey in the marinade, which is a by-product of the dairy industry. It is a good source of valuable nutrients, such as proteins, lactose, minerals (e.g., calcium and phosphorus), organic acids, and vitamins [4]. It is also a good source of microorganisms including LAB strains, with probiotic properties, and a reservoir of a number of microbial metabolites with bacteriostatic and bactericidal properties (e.g., lactic acid or bacteriocins), which support salt and other factors affecting the safety of meat products [5]. The use of acid whey for marinating meat is technologically advantageous and yields quality product [6,7,8]. In the previous work [9] we showed using in silico study the potential of beef products with acid whey to release the bioactive peptides with health-promoting potential in particular ability to act as angiotensin converting enzyme inhibitor (ACE I, 449) and dipeptidyl peptidase IV inhibitor (DPP IV, 452) but also antioxidant agent [9]. Moreover, we proved using in vitro tests that low (<3.5 kDa) and high (>3.5 kDa) molecular weight peptides could be generated in uncured roasted beef. Low molecular weight peptides have strong antioxidant activity (15.21–24.86 mg Trolox equivalents/mg peptide); this gives them their capacity to inhibit the formation of lipid peroxides (thiobarbituric acid reactive substances; TBARS) <3.0 mg malondialdehyde (MDA/kg) and reduces discoloration of the meat during the storage period [8]. Nevertheless, marinating beef in acid whey (24 h) does not significantly affect the number of peptide fractions which according with the peptidomics and bioinformatics approaches used in previous study [9] have great antioxidant activity (268 bioactive sequences) present in the final product [8]. Therefore, in this study, we aimed to determine the effect of prolonged marination time (48 h) in acid whey on the physicochemical characteristics of the products and the antioxidant activity of peptides extracted from organic dry-fermented beef.

## 2. Materials and Methods

### 2.1. Sample Preparation

In this study, we used *semimembranosus* muscle obtained from Limousine cattle (live weight of about 400–450 kg, organic breeding system, (Dukla, Poland). After 48 h of slaughter, *semimembranosus* muscles were divided into three groups. Two of these were marinated in acid whey (1.5 L) for 24 h (S1) or 48 h (S2) at 4 °C, respectively. We also maintained a control sample (without the use of acid whey in the marinade). After marination, all beef samples were salted with sea salt (3.0% (*w/w*)) and left for 24 h at 4 °C. Then, 0.8% of glucose was added to the meat. The samples were kept at 16°C and relative humidity of 75–85% for a period of 31 days for ripening. After this, the samples were cold-smoked for 1 h at 26 °C with oak-alder wood chips and then vacuum packed and stored at 4 °C. After the process of ripening (31 days) and cold storage (62 days), the samples were tested for peptide content and their antioxidant activity. Fresh acid whey was obtained after the production of cottage cheese from a local organic dairy processing plant. The properties of acid whey have been presented in a previous publication [6]. Both meat and acid whey were obtained from business entities that were certified as organic by the Polish certifying body, as per the Council Regulation (EC) No. 889/2008on organic production and labeling of organic products.

### 2.2. Extraction and Identification of Peptides

Peptides were extracted based on the method described by Mora et al., [10] after ripening (31 days) and storage (62 days). The extracts were concentrated in an evaporator and the concentrate was dissolved in 25 mL of 0.01 M HCl and filtered through a 0.45 µm membrane. Peptide contents were evaluated by measuring the content of primary amino groups (−NH_2_) according to the TNBS method described by Adler-Nissen [11], and the results were expressed as mg/mL of l-leucine amino equivalent based on the calibration curve. Next, samples were transferred to new tubes and stored at −60 °C until further analysis.

### 2.3. Electrophoretic Analysis

The extracts from fermented beef products were analyzed using sodium dodecyl sulfate-polyacrylamide gel electrophoresis (SDS-PAGE according to the method described by Laemmli [12]. Separating gel (14%) and stacking gel (5%) were used. A sample volume of 20 μL was loaded onto the gel. Electrophoresis was conducted at a constant current of 50 V for the stacking gel and 100 V for the separating gel using a Mini-PROTEAN^®^ Tetra Cell (Bio-Rad Laboratories, Hercules, CA, USA). After the destaining process, the protein degradation products were identified according to their molecular weights estimated from their relative electrophoretic motilities compared to the molecular weight standards. The amounts of each peptide band were calculated against the optical density using a Gel Doc^TM^ EZ densitometry scanner imaging system and Image Lab software (Bio-Rad, Hercules, CA, USA). The intensity of each band was calculated as its actual intensity relative to the intensity of the 15 kDa band in the prestained calibration marker (termed relative quantity) [13,14].

### 2.4. Chromatographic Analysis

The peptides were separated based on the method described by Kęska et al. [9]. The analysis was carried out after 62 days. Venn diagrams were applied to analyze the similarity of peptides from each batch [13,14].

### 2.5. Determination of Antioxidant Activity

Free radical-scavenging activity was determined by the ABTS [2,2′-azino-bis-(3-ethylbenzothiazoline-6-sulfonic acid)] method according to the method described by Re et al. [15]. Samples (100 μL) were incubated with 1000 μL ABTS. The absorbance was measured at 700 nm. Phosphate buffered saline (pH 7.4) was used as a reference sample. Percentage inhibition of the formation of ABTS radical was calculated using the following equation:

Scavenging % = [1 − (*A*_1_/*A*_2_)] × 100 at 740 nm (1)

where *A*_1_ is the absorbance of the sample and *A*_2_ is the absorbance of the control (ABTS solution). The results were expressed as IC_50_ (peptide concentrations required to inhibit the 50% of the formation of radicals). It was calculated by plotting a linear regression of percentage activity versus sample concentration, and the results are expressed as µg/mL.

The RP of the sample was determined based on the method described by Oyaizu [16]. The absorbance of the reaction product was read at 700 nm. A higher value of absorbance indicates stronger RP.

TBARS were determined according to the method described by Pikul el al. [17]. The value is expressed in terms of formation of MDA in milligrams, and it was calculated using the following equation:

TBARS (mg MDA/kg sample) = 5.5 × absorbance at 532 nm(2)

A UV–VIS spectrophotometer (U-5100 UV–Vis. Hitachi High Technologies America Inc., Schaumburg, IL, USA) was used to measure the absorbances.

ORP was measured as described by Nam and Ahn [18] using a pH meter (CPC-501; Elmetron, Zabrze, Poland) set to the millivolt scale and equipped with a redox electrode (ERPt-13, Elmetron).

### 2.6. Determination of Color Stability

Color measurements were taken using an X-Rite Color 8200 spectrophotometer (X-Rite Inc., Grand Rapids, MI, USA). The results were determined based on the CIE L*a*b* scale following the calculation of lightness (L*), redness (a*), and yellowness (b*). The measurements were performed at room temperature at 10 different points on the surface of the freshly cut meat slices with a thickness of 10 mm. Oxygenation index (ΔR) was determined as the ratio of reflectance between 630 nm (maximum of the oxygenated MYG) and 580 nm (maximum of the oxidized MYG) [6]. 

### 2.7. Statistical Analysis

All results are expressed as mean ± standard deviation (SD) of six determinations. Differences between the means of each group were assessed by two-way analysis of variance using Statistica^®^ 13.1 software (StatSoft, Poland).

## 3. Results 

### 3.1. Peptide Profile

Table 1 shows the changes in the level of peptides after 31 days of ripening and 62 days of storage. The total content of the peptides increased from an average of 1.23 mg/mL immediately after ripening (1.18 ± 0.05 mg/mL for C; 1.21 ± 0.17 mg/mL for S1 and 1.29 ± 0.05 mg/mL for S2) to about 4.26 mg/mL after the period of cold storage (4.73 ± 0.51 mg/mL; 3.95 ± 0.22 mg/mL and 4.10 ± 0.48 mg/mL for C, S1, and S2, respectively) (Table 1). The total content of peptides in the dry-fermented beef increased (between 31 and 62 days) during the cold storage (4 °C) under vacuum. Nevertheless, in marinated samples (S1 and S2), these levels were statistically and significantly lower than that of the control batch (*p* > 0.05).

**Figure 1:**
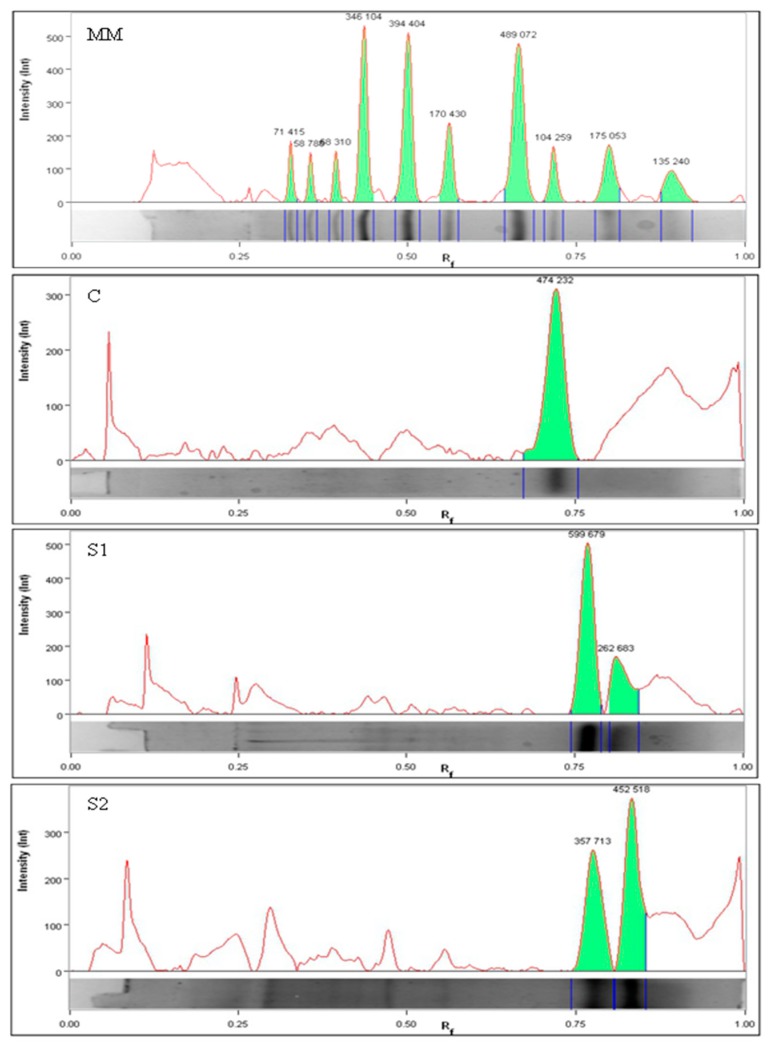
Representative densitogram of peptide extracted after fermentation (MM—molecular mass; C—control batch; S1 and S2—samples marinating for 24 h and 48 h, respectively.

Peptides from organic dry-fermented beef were profiled by electrophoresis, and the obtained results were evaluated densitometrically (Figure 1). The relative quantification of the peptides was conducted using the intensity of the ratio of bands for the respective peptides to the band intensity for the marker used (Table 2). The largest peptides were recorded for the control batch after 31 days of processing—on an average about 14.73 kDa. However, smaller peptides were recorded for S2—around 10.07 kDa.

### 3.2. Peptide Composition

**Figure 2:**
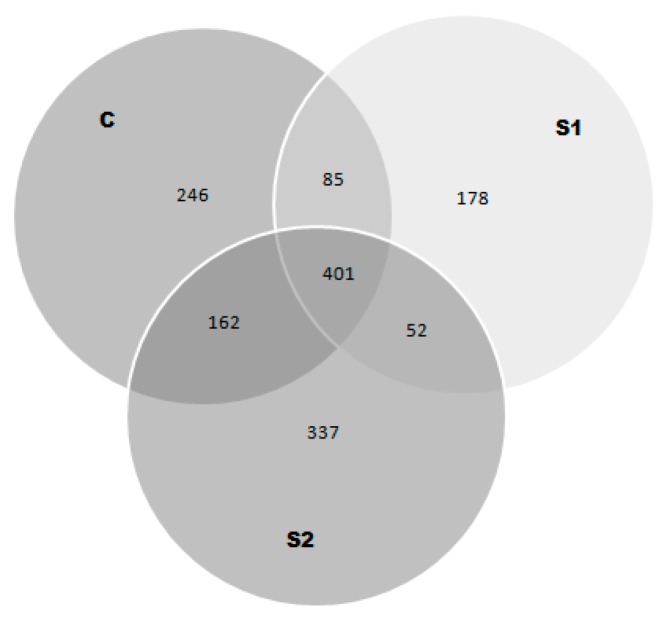
Venn diagram obtained for peptides extracted from fermented beef after refrigerated storage. C—control sample; S1 and S2—sample with marinating treatment by 24 h and 48 h, respectively.

The number of identified peptides which were common or specific to each sample were presented using Venn diagrams (Figure 2). The diagrams show that around 401 peptides were common to all samples. However, the least common sequences were recorded among samples subjected to pretreatment with acid whey (S1 and S2). The S2 sample demonstrated the presence of 337 individual peptide sequences after the treatment. 

The number of peptides obtained was not related to the molecular weight of the protein. The identified peptides were projected onto the proteins, and Table 3 shows one such sequence as an example. Supplementary data (Table S1) shows a general collection of proteins with a high sequence coverage factor for the identified peptides. To the best of our knowledge, there is no information regarding the grouping of meat proteins following the effect of acid whey. In this study, we obtained information on the number of hydrolysis products and/or on the bioavailability of specific proteins in connection with the marination process. Table 4 shows the protein’s sequence coverage based on the peptides and a selected result (quantitatively the highest amount of the peptides obtained from individual proteins). Regardless of the marinating treatment, the majority of peptides were obtained from the myoglobin (MYG) molecule, where 99% sequence coverage was recorded with the highest number of peptides reported in marinated variants (206 for S1 and 212 for S2 and 162 for C) (Table 4).

We observed a similar tendency for peptides from beta-enolase (ENOB) and triosephosphate isomerase (TPIS), as well as for peptides from actin (ACTS) and troponin (TNNT3). A high degree of percentage coverage was also reported for the aforementioned proteins in samples belonging to S2 batch (Table 4). 

### 3.3. Determination of Antioxidant Activity

Based on the results, there were differences in the antioxidant activity of the peptide fractions which were dependent on the treatment (*p* < 0.05) and processing time (*p* < 0.05).

Table 1 shows the antiradical potential of peptides extracted from organic dry-fermented beef after 31 and 62 days of processing. In the case of antiradical activity, the lowest IC_50_ value means better radical scavenging property of the peptide. On day 31 of processing, the highest value for ABTS antiradical activity was obtained for the samples from S2 batches (IC_50_ = 13.88 μg/mL), and it was almost twice as high (*p* < 0.05) as that of the remaining samples (C; S1). After 62 days of processing, the antiradical potential of peptides decreased, with the worst properties demonstrated by the samples belonging to control batches (IC_50_ for C = 23.05 μg/mL, *p* < 0.05). The S1 and S2 samples showed an average IC_50_ of 19.50 μg/mL).

After maturing in the fermentation chamber (31 days), S1 batches showed the highest RP index (RP = 1.19) and C showed the lowest (RP = 0.97; *p* < 0.05). After 62 days of processing time, the samples showed higher values in all the analyzed variants, whereas control samples showed the highest increases (by 0.81 units; *p* > 0.05). The higher the TBARS index, the more advanced will be the oxidation process in the product. After 31 days of ripening, the lowest value was recorded for S1 samples (2.03 mg MDA/kg) and the highest for the control samples (2.35 mg MDA/kg); however, the differences were not statistically significant (*p* > 0.05). After 62 days of processing, the TBARS index value decreased by about 13% in control samples, whereas in S1 and S2, it decreased by 20% and 14%, respectively. Thus, samples marinated with acid whey showed lower content of fat oxidation products. ORP values did not show any significant differences (*p* > 0.05) between the tested batches after 31 days of ripening (Table 1). The values ranged from 250.79 mV (for S1) to 258.52 mV (for S2) during this time. In all samples, ORP increased by approximately 50 mV after 62 days of processing. The greatest increase in the ORP value was recorded for samples belonging to control batch (approximately 100 mV), and this was significantly lower (*p* < 0.05) in S1 and S2 batches.

Table 1 shows the results of color parameters for each of the analyzed product. Statistical analysis showed that lightness (L*) was significantly affected by the treatment (*p* < 0.05) and storage time of the product (*p* < 0.05), whereas a* and b* values were not affected by the storage time (*p* > 0.05). The values of L* component were significantly reduced in all the tested variants (*p* < 0.05) along with the time of processing (almost 3 units for the control sample and an average of 6 units for S1 and S2 samples compared to the values measured immediately after the ripening stage (31 days). This result is in agreement with those of previous reports [6,19].

## 4. Discussion

### 4.1. Peptide Profile and Composition

The sample obtained after 48 h (S2) of marination showed the highest amount of total peptide (*p* < 0.05) in the first test period (31 days). Furthermore, 24 h of marination (S1) showed no effect on the content of peptides in relation to the control batches (*p* > 0.05); this coincides with our previous report [8]. The breakdown of proteins to peptides and amino acids is caused by the biochemical processes occurring during the process of marination and ripening, where the exogenous and endogenous enzymes are directly involved in the hydrolysis of proteins. Castellano et al. [20] and López et al. [21] reported that peptides are partly produced by the hydrolysis of the meat protein caused by the microbial enzymes. Rzepkowska et al. [5] showed that organic acid whey contains a greater number of microorganisms, among which LAB, and especially *Lactobacillus* strains, were the predominant microflora. The presence of lactose and glucose supplements may be a factor triggering the action of LAB. Moreover, pretreatment of the meat by marinating in the LAB-rich acid whey acted as an additional source of enzymes to obtain more peptides and free amino acids in organic dry-fermented beef products. The action of LAB on the meat matrix probably resulted in the better colonization and better activation of enzymatic mechanisms of LAB, including proteases. Therefore, beef proteins were additionally hydrolyzed by extracellular proteases from LAB, resulting in an increase in the groups that can be determined quantitatively by the trinitrobenzene sulfonic acid (TNBS) method after fermentation (Table 1).

The analysis of the peptide profile showed the effect of marination time on the degree of protein degradation (Table 2), which separated into two distinct clearly marked bands in these samples (S1 and S2). Based on the analysis of densitograms (blackouts expressed numerically as intensity (Int)), S2 samples showed the largest number of the smallest peptides. In addition, the intensity of these bands indicates a difference in the degree of proteolysis between the different treatments (24 h or 48 h). It is probable that the prolongation of marination time affected the activity of proteolytic enzymes, where a larger number of smaller peptides were released. Their detection was limited or even impossible due to the limitations of the separation method used (low molecular weight peptides and possibly the amino acids escaped detection by the method of electrophoresis. This shows an increased amount of protein degradation, which might be due to the action of LAB from acid whey. The pattern of peptides analyzed is a direct result of the changes in the enzyme activities that took place in the fermented products during the processing for over 62 days. The naturally generated peptides in the organic dry-fermented beef were analyzed by performing liquid chromatography-tandem mass spectrometry (nLC-MS/MS) to identify their sequences and their protein origin. Similar to other studies, in this study, the obtained individual peptide sequences for protein identification and quantification were used, and the percentage of each protein sequence covered by matching peptides was also detected [22,23,24]. The high degree of percentage coverage for MYG and HBA by the identified peptides suggested the high level of availability of these proteins for the action of proteases. Proteolytic degradation of MYG caused by the endogenous peptidases in the muscle tissue has been recorded in *postmortem* aging [25,26,27]. This result also agrees with the increased amount of hydrolysis observed in pork meat inoculated with *Lactobacillus* strains, as well as in other fermented products [20,27,28]. In turn, Gallego et al. [14] showed that the level of MYG in dry-cured pork ham showed a 10-fold decrease after the salting stage. Creatine kinase (KCRM), for which the percentage degree of coverage ranged from 52 (S2) to 64 (S1), was the most strongly hydrolyzed sarcoplasmic protein. It is involved in the energy metabolism of tissues, including skeletal muscles. Previously, various fragments of KCRM have been extracted and identified during *postmortem* aging in bovine *M*. *longissimus* *dorsi* [29]. The degradation of KCRM also has been recorded during the ripening of semi-dry-fermented sausage [17] and in the Spanish dry-cured ham [30]. Other proteins, such as ENOB or glyceraldehyde-3-phosphate (G3P), have also been detected as dominant fractions and have been shown to display a noticeable amount of proteolysis in both fermented and nonfermented meat [20,21,31]. In this study, the high degree of coverage and a large number of peptides detected suggest that these proteins are converted to smaller peptides, and it is highly probable that they contribute to the antioxidant properties of the peptides. In this study, most of the identified peptides originated from sarcoplasmic proteins (Table 4). In addition, Ferranti et al. [32] have demonstrated that the largest number of peptides isolated from the sarcoplasmic fraction of Bresaola was obtained by peptidomic approach. A greater variety of peptides derived from sarcoplasmic proteins were detected, which indicates their major contribution to general potential bioactivity. Nevertheless, some of the peptides were derived from myofibrillar proteins, such as actin, troponin, myosin, and myozenin-1 (these peptides were obtained from the hydrolysis of myofibrillar proteins that were extracted together with the sarcoplasmic proteins, e.g., due to changes in their hydrophobicity). Mora et al. [10] detected 27 peptides with molecular masses of 1706.80 Da to 3162.76 Da generated from troponin T in dry-cured ham, using the same extraction conditions as those presented in this study. As suggested by Morzel et al. [33], fragments of actin, troponin T, or myozenin-1 (also observed in this study) appeared as a direct result of the *postmortem* proteolysis of meat. In addition, Stoeva et al. [29] identified troponin T peptide fragments that arose during the aging of bovine muscle. The presence of small peptides from myoglobin (MYG) and hemoglobin (HBA) in pork dry-fermented sausages at similar processing times has also been found by other researchers [34,35].

In this study, part of the peptides identified (as an example—Table 3B) was divided into shorter fragments, e.g., ASQPDVDGFLVGGASLKPEFVD, ASQPDVDGFLVGGASLKPEF, SQPDVDGFLVGGASLKPE, and SQPDVDGFLVGGASLKPE. The differences between the sequences of these peptides indicate the participation of proteases which act on the N-terminal or C-terminal end of the peptides such as aminopeptidases and carboxypeptidases, during the processing stage for over 62 days. This effect is caused by the combined action of LAB and endogenous enzymes [31].

### 4.2. Determination of Antioxidant Activity

In this study, we investigated the antioxidant activity of peptides derived from the bovine *semimembranosus* muscle marinated in acid whey for 24 h (S1) and 48 h (S2). Antioxidant activity was measured by the reducing power (RP) of the compound against Fe^3+^ions and via measuring the antiradical activity against ABTS radicals. Malondialdehyde (MDA) formation was also determined by monitoring TBARS levels and we also recorded the oxidation-reduction potential (ORP; mV).

After 62 days of processing, the antiradical potential (ABTS*) of peptides decreased, with the worst properties demonstrated by the samples belonging to control batches. Lee et al. [36] obtained similar results in duck skin by-products (IC_50_ = 22.7 μg/mL). In this study, we did not obtain a statistically significant effect of marination time on the analyzed parameter (between S1 and S2, *p* > 0.05). Despite the fact that the content of peptides in marinated acid whey samples on the 31st day of ripening was similar (S1 = 1.21 mg / mL, S2 = 1.29 mg / mL), in the S2 test a significantly higher IC50 value was observed by about 7.26 tests S1. This may suggest that extending the marinating time does not significantly affect the overall content of biologically active peptides, only their property profile changes. The 24 h marinated test produces more peptides with antioxidant properties compared to the 48-h marinated test. This is confirmed by the significantly higher RP index obtained in beef S1 (1.19) compared to beef S2 (1.04). Contradictory results were observed for oxidation products of the fat. During processing, TBARS might get reduced in the product, probably when MDA binds covalently to amino acids from proteolytic reactions by the formation of covalent linkages via Schiff base formation [37] or via nitrite residues (MDA could undergo nitrosation reactions) [38]. This was observed for control and S2 samples in this study. However, samples from S1 batches showed a lower level of TBARS formation. There is no justification for the double marinating time (48 h) in the context of this parameter. The greatest increase in the ORP value was recorded for samples belonging to control batch (approximately 100 mV), and this was significantly lower (*p* < 0.05) in S1 and S2 batches. These results indicate the presence of low molecular weight peptides with antioxidant properties and the effect of suppression in the ORPs of the whole tissue as a result of marination in acid whey.

Meat contains various endogenous initiators and catalysts of oxidation, such as heme pigments, transition metal ions, and oxidation enzymes. In addition, processing and storage of meat intensify the processes related to oxidative degradation. Therefore, it is important to determine the antioxidant activity of the product. The color of the meat depends on the concentration of the pigment (MYG and hemoglobin), oxidation-reduction status, and light-scattering properties of the meat [39,40,41]. In this study, we recorded color measurements of the meat product to better interpret the role of antioxidative molecules from fermented beef. Marination with acid whey significantly resulted in color changes as observed in the samples belonging to S1 and S2 batches. We observed lightening of the color (L*) and a greater amount of disproportional red color (a*) compared to the control sample. The higher values of lightness (L*) in samples belonging to S1 and S2 batches might be due to the fermentation process which formed acidic compounds [7]. In turn, the increased amount of redness (a*) is probably due to the presence of LAB in acid whey which might be involved in the conversion of Mb (Fe^3+^) pigment to NO-MB pigment (Fe^2+^) [7]. There is no evidence for the effect of the marinating time (24 h or 48 h) on the color parameters. This result is in accordance with Cava et al. [42], who found that the value of redness did not change significantly in vacuum-packed pork dry-cured meat during 90 days of storage at 4 °C. Sarcoplasmic protein, including MYG and some enzymes that can participate in various biochemical processes, can affect the color stability of the meat [41,43]. According to Sayd et al. [44], muscles leading to darker meat showed a greater level of oxidative metabolism (e.g., brownish red coloration of oxidized metmyoglobin), as indicated by the presence of mitochondrial enzymes of the respiratory chain, hemoglobin, and chaperone or regulatory proteins. The higher content of oxidized MYG indicates a lower value of the △R factor (Table 1). Conversely, enzymes involved in the process of glycolysis were overexpressed in the group with low L* values in porcine *semimembranosus* muscle measured 36 h *postmortem* [44]. The lighter color of marinated meat can also result from the denaturation of the globin or the removal of heme from the MYG molecule. As mentioned earlier, S1 and S2samples (Table 4) demonstrated the presence of more amounts of peptide fragments from the MYG chains. The presence of glycolytic enzymes (fructose-bisphosphate A, fructose and ENOB, phosphoglucomutase-1, G3P dehydrogenase, pyruvate kinase M2, and KCRM) has been shown to correlate with the color of bovine products [43,45,46,47]. which partially agrees with our results (Table 4). Nevertheless, in this study, the stability of a*, b*, and ΔR parameters was observed between 31 and 62 days of processing. This effect of color stabilization results indirectly from the delayed oxidation of MYG, due to the lesser amount of oxidative factors. This is indicated by the inhibition of lipid oxidation (TBARS test) or is indicated by the presence of antioxidant compounds (ORP and ABTS test), which was particularly evident in S1. In samples from S1 batch, the lowest amount of MYGs was also recorded (*p* > 0.05), which confirms this finding.

## 5. Conclusions

Doubling of marinating time (from 24 to 48 h) as part of the preprocessing does not significantly affect the antioxidant activity of the extracts from organic dry-fermented beef. Peptide extracts from bovine *semimembranosus* muscle marinated in acid whey for 24 h (S1) showed better antioxidant activity than that of muscle marinated 48 h (S2). There was no evidence for the effect of the marination time (24 h or 48 h) on the most favorable color parameters from the point of view of the consumer, i.e., lightness and redness. The effect of marination time on the degree of proteolytic changes using peptidomic approaches was also demonstrated. The degradation of the proteins most sensitive to proteolytic degradation, i.e., MYG, hemoglobin, KCRM, and ENOB was observed in the samples after 62 days of processing. The prolongation of marination time intensifies the hydrolytic degradation of proteins and peptides in the final product, resulting in the loss (or it has no effect) of the antioxidant properties in organic dry-fermented beef.

## Figures and Tables

**Table 1:** Peptide content, antioxidant activity, and color parameters (L*, a*, b*, and oxygenation index (ΔR)) of organic dry-fermented beef.

Batch	Time [days]	Peptide Content [mg/mL]	Antioxidant Activity				Color Parameters			
			ABTS IC_50_ [µg/mL]	RP [A_700_]	TBARS [mg MDA/kg]	ORP [mV]	L*	a*	b*	ΔR
**C**	**31**	1.18 ± 0.05 ^Aa^	7.46 ± 0.71 ^Aa^	0.97 ± 0.07 ^Aa^	2.35 ± 0.62 ^Aa^	252.03 ± 15.95 ^Aa^	36.03 ± 2.40 ^Aa^	7.30 ±1.05 ^Aa^	5.27 ±1.58 ^Aa^	2.14 ± 0.26 ^Aa^
	62	4.73 ± 0.51 ^Ba^	23.05 ± 1.91 ^Ba^	1.78 ± 0.28 ^Ba^	2.05 ± 0.53 ^Aa^	347.77 ± 19.51 ^Ba^	33.34 ± 2.25 ^Ba^	7.33 ± 2.04 ^Aa^	5.76 ± 1.65 ^Aab^	2.11 ± 0.33 ^Aa^
S1	31	1.21 ± 0.17 ^Aab^	6.62 ± 0.21 ^Aa^	1.19 ± 0.09 ^Ab^	2.03 ± 0.70 ^Aa^	250.79 ± 8.24 ^Aa^	44.00 ±3.46 ^Ab^	9.11 ±1.42 ^Ab^	6.81 ±1.26 ^Aa^	2.21± 0.16 ^Aa^
	62	3.95 ± 0.22 ^Bb^	19.40 ± 1.72 ^Bb^	1.25 ± 0.11 ^Ab^	1.64 ± 0.68 ^Aa^	302.93 ± 20.57 ^Bb^	39.57 ±3.49 ^Bb^	9.40 ± 1.41 ^Ab^	7.66 ± 2.42 ^Ab^	2.23 ± 0.25 ^Aa^
S2	31	1.29 ± 0.05 ^Ab^	13.88 ± 1.41 ^Ab^	1.04 ± 0.13 ^Aa^	2.17 ± 0.94 ^Aa^	258.52 ± 15.72 ^Aa^	43.78 ±2.81 ^Ab^	9.01 ±2.00 ^Ab^	6.75 ±1.98 ^Aa^	2.15 ± 0.31 ^Aa^
	62	4.10 ± 0.48 ^Bb^	19.77 ± 1.34 ^Bb^	1.41 ± 0.12 ^Bc^	1.87 ± 0.91 ^Aa^	301.82 ± 12.47 ^Bb^	36.08 ± 4.67 ^Ba^	7.22 ± 1.87 ^Aa^	4.22 ± 1.75 ^Ba^	2.00 ± 0.37 ^Aa^

Note: ^A,B^ Within the same treatment, means followed by a common letter do not differ significantly (*p* < 0.05); ^a,b^ Within the same time, means followed by a common letter do not differ significantly (*p* < 0.05). ABTS, 2,2′-azino-bis(3-ethylbenzothiazoline-6-sulfonic acid); RP, reducing power; TBARS, thiobarbituric acid reactive substances; ORP, oxidation-reduction potential.

**Table 2:** Relative band intensities of samples varying with the method of treatment after 31days of processing.

Batch	Band No	Molecular Weight [kDa]	Band Intensities	Bands [%]
C	*1*	14.73 ± 0.08 ^a^	2.59 ± 0.82 ^a^	100 ± 0.00
	*2*	nd ^*^	nd	nd
S1	*1*	14.68 ± 0.37 ^a^	3.03 ± 0.96 ^a^	33.25 ± 6.96
	*2*	13.78 ± 0.16 ^b^	1.79 ± 0.79 ^a^	66.75 ± 6.03
S2	*1*	11.76 ± 0.49 ^c^	1.44 ± 0.42 ^a^	37.30 ± 6.35
	*2*	10.07 ± 0.09 ^d^	2.91 ± 0.42 ^a^	62.70 ± 6.35

The relative quantity of electrophoretic bands corresponding to the peptides extracted from the fermented beef after treatment. Spot quantity is expressed as mean values ± standard deviation. Different letters indicate significant differences between treatment for each band at *p* < 0.05 (*n* = 5); * nd—not detected.

**Table 3:** The result of the projection of the identified peptides from triosephosphate isomerase sequence (ID Q5E956) (A) and the detailed list of peptides (B). Matching peptides shown in *bold*

**(A)**	**C**	**Protein sequence coverage: 7%.**				
		10	20	30	40	50
		MAPSRKFFVG	GNWKMNGRKN	NLGELINTLN	AAKVPADTEV	VCAPPTAYID
		60	70	80	90	100
		FARQKLDPKI	AVAAQNCYKV	ANGAFTGEIS	PGMIKDLGAT	WVVLGHSERR
		110	120	130	140	150
		HVFGESDELI	GQKVAHALAE	GLGVIACIGE	KLDEREAGIT	EKVVFEQTKV
		160	170	180	190	200
		IADNVKDWSK	VVLAYEPVWA	IGTGKTATPQ	QAQEVHEKLR	GWLKSNVSDA
		210	220	230	240	
		VAQSARIIYG	GSVTGATCKE	LA***SQPDVDGF LVGGASLKPE F***VDIINAKQ		
	**S1**	**Protein sequence coverage: 8%**				
		10	20	30	40	50
		MAPSRKFFVG	GNWKMNGRKN	NLGELINTLN	AAKVPADTEV	VCAPPTAYID
		60	70	80	90	100
		FARQKLDPKI	AVAAQNCYKV	ANGAFTGEIS	PGMIKDLGAT	WVVLGHSERR
		110	120	130	140	150
		HVFGESDELI	GQKVAHALAE	GLGVIACIGE	KLDEREAGIT	EKVVFEQTKV
		160	170	180	190	200
		IADNVKDWSK	VVLAYEPVWA	IGTGKTATPQ	QAQEVHEKLR	GWLKSNVSDA
		210	220	230	240	
		VAQSARIIYG	GSVTGATCKE	L***ASQPDVDGF LVGGASLKPE FVD***IINAKQ		
	**S2**	**Protein sequence coverage: 18%**				
		10	20	30	40	50
		MAPSRKFFVG	GNWKMNGRKN	NLGELINTLN	AAKVPADTEV	VCAPPTAYID
		60	70	80	90	100
		FARQKLDPKI	AVAAQNCY***KV ANGAFTGEIS PGMIKDLGAT W***VVLGHSERR			
		110	120	130	140	150
		HVFGESDELI	GQKVAHALAE	GLGVIACIGE	KLDEREAGIT	EKVVFEQTKV
		160	170	180	190	200
		IADNVKDWSK	VVLAYEPVWA	IGTGKTATPQ	QAQEVHEKLR	GWLKSNVSDA
		210	220	230	240	
		VAQSARIIYG	GSVTGATCK***E LASQPDVDGF LVGGASLKPE F***VDIINAKQ			
**(B)**		Peptides Sequence	Mass	C	S1	S2
		SQPDVDGFLVGGASLKPE	1814.90506	-	+	+
		VGGASLKPEFVDIINAKQ	1885.0309	-	-	+
		GAFTGEISPGMIKDLGATW	1949.9557	-	-	+
		SQPDVDGFLVGGASLKPEF	1961.97348	+	+	+
		ASQPDVDGFLVGGASLKPEF	2033.01059	-	-	+
		ELASQPDVDGFLVGGASLKPEF	2275.13724	-	-	+
		KVANGAFTGEISPGMIKDLGATW	2362.19911	-	-	+
		ASQPDVDGFLVGGASLKPEFVD	2247.10596	-	+	-

**Table 4:** Identification of the selected proteins by nLC–MS/MS in organic dry-fermented beef after 62 days.

Protein Name	ID ^a^	Mass [Da]	C		S1		S2	
			% Coverage ^b^	Peptides Identified ^c^	% Coverage ^b^	Peptides identified	% Coverage ^b^	Peptides Identified
Myoglobin	sp|P02192|MYG_BOVIN	17.078	99.000	162	99.000	206	99.000	212
Hemoglobin subunit alpha	sp|P01966|HBA_BOVIN	15.184	65.000	20	46.000	10	51.000	13
Creatine kinase type M	sp|Q9XSC6|KCRM_BOVIN	42.989	59.000	113	64.000	130	52.000	108
Beta-enolase	sp|Q3ZC09|ENOB_BOVIN	47.096	45.000	49	45.000	65	56.000	65
Actin, alpha skeletal muscle	sp|P68138|ACTS_BOVIN	42.051	37.000	36	57.000	89	58.000	77
Glyceraldehyde-3-phosphate dehydrogenase	sp|P10096|G3P_BOVIN	35.868	23.000	34	36.000	39	36.000	43
Myozenin-1	sp|Q8SQ24|MYOZ1_BOVIN	31.674	23.000	6	11.000	8	11.000	9
Troponin T, fast skeletal muscle	sp|Q8MKI3|TNNT3_BOVIN	32.126	22.000	18	30.000	34	30.000	26
Phosphoglucomutase-1	sp|Q08DP0|PGM1_BOVIN	61.589	11.000	5	8.000	6	8.000	6
Myosin-1	sp|Q9BE40|MYH1_BOVIN	222.290	10.000	53	11.000	66	12.000	54
Triosephosphate isomerase	sp|Q5E956|TPIS_BOVIN	26.690	7.000	1	8.000	3	18.000	7

**Note:**^a^ Accession number according to the UniProt database. ^b^ Percentage of each protein’s sequence covered by matching peptides. ^c^ The number of peptides assigned to a given protein. Multiple modified and cleaved states of the same underlying peptide sequence are considered distinct peptides because they have different molecular formulas. nLC–MS/MS, liquid chromatography–tandem mass spectrometry.

## Supplementary Materials

The following are available online at https://www.mdpi.com/2218-273X/9/10/614/s1, Table S1: List of the peptides identified by LC-MS/MS from selected proteins in organic dry-fermented beef after 62 days.
